# Computational modelling for the sustainable management of chemicals

**DOI:** 10.1016/j.comtox.2020.100122

**Published:** 2020-05

**Authors:** Andrew P. Worth

**Affiliations:** European Commission, Joint Research Centre (JRC), Ispra, Italy

**Keywords:** Computational toxicology, Green deal, Digital age, Chemicals, Disease burden, Ecosystem damage

## Abstract

This commentary explores the contribution of computational toxicology to chemical safety assessment in the context of the broad policy challenges faced by the European Union. The state of the European Environment is considered from the perspective of chemical contributions to the burden of disease and ecosystem damage. This sets the scene for highlighting research and innovation opportunities to further develop computational approaches for assessing the human health and environmental effects of chemicals. Emphasis is placed on focus topics that are particularly relevant to the political priorities of the new European Commission. In particular, two of the six priorities are discussed - “The European Green Deal” and “A Europe fit for a Digital Age”. The former includes the zero pollution ambition for a toxic-free environment, including the need to develop safe and sustainable chemicals, while the latter includes the challenges and opportunities posed by Artificial Intelligence. This commentary is based on a presentation given at the 19th meeting of The Italian Society of Toxicology (SITOX), held in Bologna, Italy, in February 2020.

“The best way to predict the future is to create it”. This saying is attributed to the 16th President of the United States, Abraham Lincoln, and is just as much true today as it was in the 1860s. When it comes to the issue of planetary health, it also takes on a new urgency. In this article, I explore the relationship between computational modelling and the safety assessment of chemicals, and how this fits into the European Commission’s political priorities aimed at protecting people and the environment.

Firstly, the societal problem and policy challenge could not have been articulated more starkly than by the European Environment Agency (EEA) in December 2019, when it released its 5-yearly report into the state of the European environment [1]. The publication was launched at a high profile press conference by Frans Timmermans, Executive Vice-President of the European Commission, Virginijus Sinkevičius, European Commissioner for Environment, Oceans and Fisheries, and Hans Bruyninckx, Executive Director of the European Environment Agency (EEA). The report highlights that while European environment and climate policies have helped to improve the environment over recent decades, Europe is not making enough progress. As stated by Hans Bruyninckx, “Europe’s environment is at a tipping point. We have a narrow window of opportunity in the next decade to scale up measures to protect nature, lessen the impacts of climate change and radically reduce our consumption of natural resources.”

Safeguarding the environment is of course a wicked problem, with complex interdependencies between cause and effect. The extent to which chemicals in our environmental are contributing to the problem is largely unknown, but certain trends can be identified. Firstly, our knowledge about chemical properties is the tip of an iceberg. It has been estimated that there are about 100,000 chemicals on the market, out of which around 500 have been extensively studied for the health and environmental effects. Another 30,000 chemicals have been characterised to some extent, while there is little to no knowledge about the remaining 70,000 [1]. In a more recent study, 22 chemical inventories from 19 countries and regions were analysed to achieve a global overview of chemicals on the market [2]. According to this analysis, over 350,000 chemicals and mixtures of chemicals have been registered for production and use, up to three times as many as previously estimated. Whatever the actual number, the chemicals knowledge gap represents an opportunity for computational modelling to study legacy chemicals and pave the way for sustainable chemicals in the future.

Another important trend concerns the growth in the global chemicals market. Global chemical sales, excluding pharmaceuticals, are projected to roughly double from 3.47 trillion Euro in 2017 to 6.6 trillion Euro by 2030 [3]. We therefore need to ensure that the level of pollution does not double as well.

In terms of impacts on public health, there are authoritative reports on the contribution of chemicals to the global burden of disease. In 2018, the WHO estimated the disease burden preventable through the sound management of chemicals at around 1.6 million lives and around 45 million disability-adjusted life years (DALYs) in 2016 [4]. This corresponds to 2.7 per cent of total global deaths and 1.7 per cent of the total burden of disease worldwide for that year. Moreover, these figures are likely to be underestimates, given that they are based only on exposures to chemicals for which reliable data exist. This also has a substantial economic impact. It has been estimated that the economic burden on health care systems, and the lost productivity of the workforce, amounts to several percentage points of GDP [5].

There is also mounting evidence of adverse chemical effects on wildlife and ecosystems [6]. Examples include the effects of neonicotinoid pesticides on bee health, and more recently the effects of UV filters in sunscreens on the health of coral reefs. Such evidence is informing chemicals policy through bans or restrictions on certain chemicals.

Countries around the world are recognising and responding to this problem. In the European Union, the European Commission has adopted 6 political priorities, of which two in particular are relevant to the challenge of chemical sustainability. The “European Green Deal” sets out an ambitious set of actions aimed at making the EU the world's first “climate-neutral” continent by 2050 [7]. This includes the ambition of moving towards a “non-toxic environment”. The Green Deal is not only focussed on the state of the European environment, but also has an international dimension, in particular by reinforcing the EU’s commitment to the UN Sustainable Development Goals (SDGs [8]). Three of the SDGs are linked to chemicals: SDG3 on “good health and wellbeing”, SDG6 on “clean water and sanitation” and SDG12 on “responsible consumption and production”.

The EU chemicals policy has been evolving since the 1960s, and now comprises more than 40 pieces of legislation. The legal provisions in this chemicals acquis apply to specific sectors (e.g. pesticides) and environmental media (e.g. freshwater) but also work in a collective manner. For example, the cosmetics regulation ensures the human safety of cosmetic ingredients and products, but does not address possible environmental effects of cosmetics in the environment. However, this aspect is picked up by the REACH regulation, since cosmetic ingredients need to be registered under REACH. Another important regulatory synergy is between legislation that requires safety data on chemicals to be generated, such as REACH, plant protection products and biocides, and the CLP regulation that imposes no data requirements, but uses the data generated under other legislation to classify chemicals based on their intrinsic hazards. The classification and labelling then forms the basis of risk management measures such as bans and restrictions, where needed. In addition, some pieces of legislation require exposure assessments to be conducted, so that the information on hazards and exposures can be combined into a risk assessment. The risk assessment allows safe exposure levels to be determined, according to context of use.

It is also important to note that underpinning the chemicals legislation is a cross-cutting policy to ensure animal welfare, as enshrined in the Treaty on the Functioning of the EU, and Directive 2010/63 on the protection of animals used for scientific purposes. Animal welfare considerations, combined with the need to efficiently and effectively evaluate large numbers of chemicals have led to the increasing acceptance and use of computational methods in the safety assessment process.

An important element of the EU chemicals policy and Green Deal is the drive towards a circular economy for chemicals, in other words, moving away from the manufacture, (single) use and disposal of chemicals, to the recycling and re-use of chemical products. This poses a challenge, because on the one hand there is a need to re-use chemicals, but there is also a need to remove chemicals of high concern from the product supply, and to do so in an economically and environmentally viable way (i.e. without too much energy demand). This also represents an opportunity for innovation in the chemicals industry, and an opportunity to further develop and apply computational prediction tools.

Another political priority, “A Europe fit for the Digital Age” sets out measures to develop digital technologies and Artificial Intelligence, which will enable actions under the other priorities [9]. The rapidly developing field of Artificial Intelligence also provides opportunities for innovation. The computational modelling of chemical toxicity already exploits big data and “smart” machine learning algorithms [10]. Progress in this direction can only continue. Moreover, the use of AI in chemical risk assessment is likely to go beyond modelling per se, to facilitate other elements of the risk assessment process, including both technical and social aspects of the decision-making process [11]. At the policy level, guidelines are being developed for “trustworthy AI” [12]. These include principles that the modelling community is familiar with, such as the need for reliable, transparent and explainable algorithms, implemented in a secure and sustainable way. Another issue concerns privacy, especially as personalised health data are increasingly being made available, opening up opportunities to develop population-based models, capturing variations in chemical exposure and different susceptibilities to disease. At present, EU guidelines on AI are essentially of a self-regulatory nature, but there is growing demand for more government oversight [13]. European Commission President Ursula von der Leyen has stressed the need for the EU to lead the transition to a healthy planet and a new digital world. This twin challenge of a green and digital transformation has to go hand-in-hand [9].

Modelling cause-effect relationships, such as how chemicals contribute to disease and ecosystem damage, is not only a scientific endeavour, but provides a direct contribution to decision-making throughout the policy cycle. In the EU, the Commission is required to carry out an impact assessment of different regulatory options. This is sometimes called an ex ante assessment, and relies partly on the use of simulation models. Once legislation has been adopted, models play a further role in its implementation, and this is where computational models for toxicity prediction are most widely used. Different kinds of computational model are used at different steps of the risk assessment process, based on exposure modelling and hazard modelling. There is an increasing and bewildering array of digital resources for modelling, including data bases and predictive tools [14]. Finally, the EU carries out periodic reviews on the effectiveness and efficiency of existing legislation. This is called an ex post evaluation or fitness check. In this context, other kinds of evidence (such as cost-effectiveness analysis) are typically used. A fitness check of chemicals legislation is a very challenging exercise, partly due to the difficulty in establishing causal relationships between chemicals and their impacts on health and the environment. In this context, it is also a challenge to evaluate the extent to which legislative action has reduced or prevented harm.

The Green Deal and other policy documents do not specify what kinds of models need to be developed for what purposes. However, opportunities for modellers to innovate can be taken from some of the big challenges. Thus, several focus areas for modelling can be identified: the combined effects of mixtures, non-communicable diseases, endocrine disruptors, and chemicals that are persistent and bioaccumulative.

Non-communicable diseases such as Parkinsons and other neurodegenerative disease are notoriously difficult to model, and this is partly because of long time-lag between chemical exposure and the onset of disease, as well as interactions between different chemicals and other stressors (the combined exposure challenge). A promising and emerging approach however is the modelling of networks of Adverse Outcome Pathways, which capture the multitude of ways chemicals can interact with the body and perturb biological systems from a healthy state to disease [15]. Of course, chemical risk assessments informed by modelling are only a partial solution to the problem. The modelling needs to embedded in a more holistic approach. For example, cancer prevention depends partly on the identification of chemical carcinogens, but also on efforts to focus on the most prevalent cancers and the most susceptible individuals in the population [16].

Another challenge for chemical risk assessment concerns possible impacts on ecosystems. There are simply too many chemicals, and too many species to take into account. Here again, modelling offers a way forward [17]. For example, it is known that some species are most sensitive to others to the toxic effects of chemicals, and it is possible to extrapolate from the effects on one species to the effects on another. Thus, it is not necessary to measure effects in all species of concern. Similarly, it is not necessary to test all chemicals on a given species, since it is known that a log-normal distribution defines the relationship between the most toxic and the least toxic chemicals. This means that it is only necessary to model a pre-defined percentile of the toxicity distribution, such as the 5th percentile. This approach is called the Threshold of Toxicological Concern, and is a powerful means of making decisions on the basis of historical data [18].

In conclusion, the environmental challenges of our time are unprecedented. Business as usual is not an option, since to paraphrase UN Secretary General Ban Ki-Moon, there is “no planet B”. The sustainable management of chemicals requires innovation at multiple levels: innovation in policy making, risk management and risk assessment. This means there are multiple opportunities for sustainable chemistry, including modelling. Finally, all of this will depend on the ability to connect the dots, build bridges across different knowledge disciplines [19], and to do so in a creative way.

## Declaration of Competing Interest

The authors declare that they have no known competing financial interests or personal relationships that could have appeared to influence the work reported in this paper.
